# Using *Thymus carmanicus* and *Myrtus communis* essential oils to enhance the physicochemical properties of potato chips

**DOI:** 10.1002/fsn3.597

**Published:** 2018-04-16

**Authors:** Leila Sedaghat Boroujeni, Mohammad Hojjatoleslamy

**Affiliations:** ^1^ Department of Food Science and Technology Science and Research branch Islamic Azad University Tehran Iran; ^2^ Department of Food Science and Technology Islamic Azad University Shahrekord branch Shahrekord Iran

**Keywords:** antioxidant, essential oil, lipid oxidation, *Myrtus communis*, potato chips, *Thymus carmanicus*

## Abstract

The aim of this study was to evaluate the antioxidant effects of the essential oils of *Myrtus communis* leaves and *Thymus caramanicus* aerial parts in order to improve the physicochemical properties of potato chips. Sunflower oil without any antioxidant (control group) was fortified with BHA or TBHQ antioxidants (200 ppm), and *M. communis* or *T. caramanicus* essential oils (3,000 ppm). The effects of the antioxidant behavior of these compounds on the physicochemical properties of potato chips were analyzed by measuring peroxide value (PV), acid value (AV), and thiobarbituric acid (TBA). In addition, changes occurring in oxidation stability, texture, and color were evaluated. The results revealed that samples containing either *M. communis* or *T. carmanicus* showed a significant decrease in PV, as compared to the control sample. Compared with the control, the extracted oil of potato chips with *M. communis* or *T. carmanicus* led to the significant reduction in AV (*p* < .05). The results also revealed the addition of the essential oils of *M. communis* or *T. carmanicus* was obviously effective in preventing the TBA increasing value. Based on the results obtained by the Rancimat test, either *T. carmanicus* or *M. communis* essential oils could significantly increase the shelf‐life of potato chips, as compared with the control sample (*p* < .05). The hardness of potato chips was decreased in *M. communis* or *T. carmanicus* groups (*p* < .05), as compared to the control sample. Neither *M. communis* nor *T. carmanicus* essential oils had any negative effects on the lightness values, as compared to the control (*p* > .05). Based on the results, the physicochemical properties of potato chips could be improved with the addition of these essential oils.

## 1 INTRODUCTION

Dietary lipids, which are naturally occurring in raw food materials or added during food processing, serve a significant role in food industries. Lipid oxidation is the main cause of food quality deterioration; it has been a challenge for food manufacturers. Lipids may undergo oxidative processes in the presence of oxygen, moisture, light, heat, microorganisms, or the action of enzymes, leading to the loss of food wholesomeness by the deterioration of aroma and flavor, as well as the decay of nutritional value and food safety qualities (Allen & Angelo, 1992; Shahidi, 2000).

The main purpose of using an antioxidant, both natural and synthetic, as an additive in the food industry is to maintain quality and prolong the shelf‐life of the foodstuffs by protecting them against the deterioration caused by oxidation (Wanasundara & Shahidi, 2005). A variety of natural and synthetic antioxidants are used in fat‐containing foods in the industry; the majority of these are synthetically manufactured. By focusing on the safety issue of these ingredients, a large body of research has identified unwanted problems of these synthetic antioxidants for humans, such as carcinogenic effects (Race, 2009). Some of the most frequently synthetic groups of antioxidants used as food additives include tertiary‐butyl hydroquinone (TBHQ), butylated hydroxyanisole (BHA), and butylated hydroxytoluene (BHT; E319‐E321) group. This group is used in a variety of products, but it is most commonly found in foods that contain oil and fat (Wanasundara & Shahidi, 2005). BHA and BHT have been used in food products with some restrictions since the late 1950s; also, in Europe, TBHQ became an accepted antioxidant for food use in 2004. These prohibitions result from unsafe effects reported by some studies (Race, 2009; Weber, 2014; WHO, 1998). Consequently, there is an increasing tendency to use naturally occurring antioxidants in food products, in order to replace the more conventional synthetic antioxidants (Wanasundara & Shahidi, 2005).

Essential oils are among the well‐known compounds, and their beneficial effects on human health have been widely studied and established (Bakkali, Averbeck, Averbeck, & Idaomar, 2008). Therefore, the use of these as antioxidative agents in food systems may be considered to increase the safety, quality, and the useful shelf‐life of foods, especially fats or oils subjected to frying. Fried potato chips, one of the most popular foodstuffs, are consumed in large quantities all over the world (Thakur & Saxena, 2000). The efforts made to develop functional foods have been increased in recent years. These food products provide a positive impact on human health, in addition their nourishment property; these effects can help to decrease the heart diseases risk, cardiovascular problems, and diabetes (Hasler, 2002).

*Myrtus communis* L. is one of the important aromatic and medicinal species belonging to the family Myrtaceae. *M. communis* has a long history of application in the cosmetic, pharmaceutical, and food industries (Aleksic & Knezevic, 2014). An extensive literature survey has revealed that *M. communis* has a long history of traditional use for a variety of diseases; many of these effects have been validated by scientific research (Aleksic & Knezevic, 2014). Several reports describe the antioxidant properties of different extracts and compounds obtained from *M. communis* (Rahimmalek, Mirzakhani, & Pirbalouti, 2013; Yadegarinia et al., 2006; Aleksic and Knezevic, 2014; Wannes et al., 2010; Serce, Ercisli, Sengu, Gunduz, & Orhan, 2010). Thymus (thyme), with the common Persian name of “Avishan,” is an aromatic plant belonging to the family Lamiaceae (Zargari, 1990). *Thymus caramanicus* Jalas is one of the species of Thymus that is an endemic Iranian species and grows in different regions of Iran (Zargari, 1990). The essential oils and extracts of Thymus species are commonly used in pharmaceutical, cosmetic, and perfume industries for a number of products (Bauer, Garbe, & Surburg, 1997). *Thymus* species are well‐known as medicinal plants because of their biological and medicinal properties. Previous studies have demonstrated that *Thymus* species have antioxidative activities (Bozine, Mimica‐Dukic, Simin, & Anackov, 2006; Giorge et al., 2010; Hayder et al., 2008; Safaei‐Ghomi, Ebrahimabadi, Djafari‐BidgoliZ, & Batooli, 2009).

According to the mentioned data, this study aimed to investigate the possible effects of essential oils from *M. communis* and *T*. *caramanicus* on improving oil oxidation and also, the characteristics of fried potato chips. This study was therefore undertaken to:

(1) evaluate the chemical composition of *M. communis* leaf and *T. carmanicus* aerial part essential oils; (2) study the antioxidant effects of the addition of these essential oils on the oil oxidation of potato chips, and compare these with the synthetic antioxidants BHA and TBHQ; and (3) evaluate the effects of these essential oils on some physicochemical properties of potato chips.

## 2 MATERIALS AND METHODS

### 2.1 Plant materials and essential oil extraction

Leaves of *M. communis* were collected from Maadan region (located in Chaharmahal and Bakhtiari Province, Iran), and *T. carmanicus* aerial parts (consisting of stems, leaves, and flowers) were purchased from a market in Kerman, Iran. The plants were identified using a valid botanical reference (Zargari, 1990). Voucher specimens had been kept in the Herbarium of I.A.U. Shahrekord Branch (no. 231). The plant materials were dried in shadow at room temperature for 5 days; then, they were ground in an electric grinder into pieces of 0.5–1 cm. One hundred grams of each powdered sample was weighed and submitted to water distillation for 3 hr, using a Clevenger‐type apparatus, according to the method recommended by European Pharmacopoeia (Council of Europe, 1996). The obtained essential oils were dried over anhydrous sodium sulfate and stored in dark glass bottles at 4°C until use.

### 2.2 Gas chromatography‐mass spectrometry (GC‐MAS) identification

The GC analyses were carried out on an Agilent Technologies 7,890 gas chromatograph (Agilent Technologies, Santa Clara, CA, USA) equipped with a single injector and a flame ionization detector (FID). An apolar HP‐5 capillary column (30 m × 0.25 mm ID, 0.25 μm film thickness) coated with 5% phenyl and 95% methyl polysiloxane was used. The carrier gas was nitrogen with the flow rate of 0.8 ml/min. The initial column temperature was 60°C; it was programmed to increase at 4°C min^−1^ to 280°C. The injector temperature was set at 280 and 300°C. Split injection was conducted with a ratio split of 1:40. Essential oil samples of 0.1 μl were injected neatly. GC‐MS analyses were performed on an Agilent Technologies 7,890 gas chromatograph coupled to the Agilent 5,975°C mass selective detector (MSD) and the quadrupole EI mass analyzer (Agilent Technologies, Palo Alto, CA, USA). An HP‐5MS 5% column (30 m × 0.25 mm, 0.25 μm film thickness) was used as the stationary phase. Helium was used as the carrier gas at 0.8 ml/min flow rate. The temperature was programmed from 60°C to 280°C at the 4°C min^−1^ ramp rate. The injector and the GC‐MS interface temperatures were maintained at 290°C and 300°C, respectively. Mass spectra were taken at 70 eV, and the mass range was from *m/z* 50 to 550 amu. The components were identified based on the comparison of their mass spectra with those of NIST08 (National Institute of Standards and Technology) and Willey (ChemStation data system) libraries, as well as by comparison with the calculation of their retention indices, as reported in the literature (Adams, 2001). The relative proportions of the essential oil constituents were expressed as percentages obtained by the peak area normalization.

### 2.3 Frying oil and potato chips preparation

Frying sunflower oil without any antioxidant was obtained from Nahangol Co. (Nahangol, Boroujen, Iran), and the profile of the fatty acids of sunflower was developed using a gas chromatograph equipped with a flame ionization detector (GC‐FID), as shown in Table 1. Three most abundant fatty acids in sunflower oil used in this study were in the order: linoleic acid (58.63%), oleic acid (27.96%), and palmitic acid (7.62%). Pretests had been performed by different concentrations of essential oils, but 3,000 ppm showed the best antioxidant activity which could be compared to 200 ppm synthetic antioxidants. Sunflower oil had been selected due to its extensive use. The essential oils of *M. communis* and *T. carmanicus* were added in the concentration of 3,000 parts per million (ppm), and synthetic antioxidants BHA and TBHQ, each with levels of 200 ppm, were added to the sunflower oil (without any antioxidant) in a dark‐colored glass. Also, an oil sample without any additives was prepared as the control group. These samples were used to determine the antioxidant effects of essential oils on the potato chips properties. Agria potatoes were prepared from Zarin Tapesh Khavaran Co. (Boroujen, Iran). Potatoes were washed, peeled, and sliced (1.3 mm thickness) using a sterile stainless steel slicer. Slices were rinsed immediately after cutting in distilled water until the frying process. Potato slices were transferred to the fryer (ADR2, Moulinex, France) containing hot sunflower oil; they were fried for 13 ± 2 min. Frying temperature was kept almost constant (±1°C). All samples were packed in aluminum bags and stored in an electric oven at 60 ± 1°C for a storage period of 60 days to investigate their physicochemical properties.

**Table 1:** Fatty acids composition of consumed sunflower oil

Fatty acid name	Fatty acid	Quantity (%)
C18:2 *cis*	Linoleic acid	58.63
C18:1 *cis*9	Oleic acid	27.96
C16	Palmitic acid	7.62
C18	Stearic acid	4.25
C18:3 *cis*	Linolenic acid	0.54
C18:2 *trans*	Linoelaidic acid	0.25
C20	Arachidonic acid	0.25
C20:1	Gondoic acid	0.18
C16:1	Palmitoleic acid	0.09
C14	Myristic acid	0.09
C20:2	EicosaDienoic acid	0.08
Total		**99.94**

### 2.4 Rancimat test

The oxidation stability of oil samples was studied in the experimental conditions. The impact of the antioxidative properties on the stability of sunflower oil was compared to that of the control (without any antioxidant) to determine oxidation induction time (hr), as measured by the Rancimat apparatus (Metrohm 743, Switzerland). About 3 ml of each sample was placed in the heating block device. The temperature was set at 120°C. The electrodes were placed in 60 ml of distilled water, and the airflow was set at 20 L/hr. The air supply was connected to the tubes containing the oil samples, and the chart recorder was started. Over time, the volatile oxidation products were trapped in distilled water and determined conductometrically. The induction time was defined as the necessary time to reach the inflection point of the conductivity curve (Halbault, Barbé, Aroztegui, & De La Torre, 1997).

### 2.5 Determination of the antioxidant effects of different oil samples on the physicochemical properties of potato chips

The oil samples were extracted from potato chips after frying; then, peroxide value (PV), acidity value (AV), and thiobarbituric acid (TBA) were measured. Physical properties of the fried potato chips, including color and texture parameters, were also evaluated.

#### 2.5.1 Peroxide value (PV) and acidity value (AV)

The potato chips were packaged and kept in an electric oven at 60 ± 1°C for 60 days; the variations in PV and AV of the extracted oils from stored potato chips were determined according to the AOCS methods (A.O.C.S, 1998) on days 15, 30, 45, and 60. The PV was reported as milliequivalents of oxygen per kilogram of sample (meq/kg).

#### 2.5.2 Thiobarbituric acid (TBA) value

One gram fat of each of the extracted oils from the stored potato chips was dissolved in 10 ml of carbon tetrachloride and 10 ml of thiobarbituric acid was added to it; then, it was centrifuged at 250 g for 5 min, and the supernatant was separated and placed in the water bath for 30 min; after that, the absorbance of reactive substances to TBA was measured at 530 nm. TBA value was expressed as milligrams of malondialdehyde (MDA) equivalents per kilogram sample or as micromoles of MDA equivalents per gram of sample.

TBA was calculated according to equation 1:

(1) $$E_{1\mathrm{cm}}^{1g}=\frac{e}{d.a}$$

where *e* is the measured optical absorbance, d is the optical cell thickness (cm), and *a* is the sample weight (g).

### 2.6 Color measurement

The color values of the samples were determined using a tristimulus colorimeter (Hunter Lab Color flex EZ, USA). Results were the average of ten measurements taken at room temperature.

ΔE (total color change) was calculated for the samples by the equation 2:

(2) $$\Delta E=\surd {\left(L_{0}^{*}-L^{*}\right)}^{2}+{\left(a_{0}^{*}-a^{*}\right)}^{2}+{\left(b_{0}^{*}-b^{*}\right)}^{2}$$

L* (±brightness), a* (±green‐red), and b* (±yellow‐blue).

Browning Index (BI) was determined using equations 3 and 4 (Bal, Kar, Satya, & Naik, 2011; Cefola et al., 2012):

(3) $$\text{BI}=(100^{*}\left(X-0.31\right))/0.17$$

(4) $$X=\left({\text{at}}^{*}+1.75\;L^{*}\right)/\left(5.645\;L^{*}+a0^{*}\_3.012\;{\text{bt}}^{*}\right)$$

### 2.7 Texture analysis

Breaking force of the samples was measured by the puncture test, using a texture analyzer (CT3, Brookfield engineering, USA). One chip of potato for each treatment (control, fried by enriched oil with each of TBHQ, BHA, *M. communis* essential oil, or *T. carmanicus* essential oil) was placed on the sample holder and punctured with a stainless probe (No. 44) at the ambient temperature. At least ten samples were developed in each assay; and in each test, the breaking force was measured.

### 2.8 Statistical analysis

All extractions and determinations were conducted in triplicates. The data were statistically analyzed by employing a completely randomized design (CRD), using SPSS (version 18.0) software. Means of the main constituents of the essential oils were compared by Duncan's multiple range test at *p* < .05 level.

## 3 RESULTS

### 3.1 Chemical composition of the essential oils

The composition of the essential oils was analyzed by GC‐MS, the general chemical profiles of *M. communis* leaves and *T. carmanicus* aerial parts essential oils, the percentage content of the individual components, and retention indices, as summarized in Tables 2 and 3, respectively. The oil yields were 1.6 ± 0.1% and 0.56 ± 0.1% (v/w) on a dry weight basis for *T. carmanicus* and *M. communis*, respectively.

**Table 2:** The essential oil components of *Myrtus communis*

Compound	RI	Content (rel. %)
α‐Thujan	929	0.66
α‐Pinene	939	41.55
Camphene	950	0.11
β‐Pinene	978	0.83
β‐Myrcene	991	0.27
α‐Phellandrene	1,006	0.21
∆‐3‐Caren	1,011	0.38
α.‐Terpinene	1,017	0.14
1,8‐Cineol	1,034	32.24
Β‐Ocimene Y	1,036	0.18
Β‐Ocimene Y	1,046	0.4
γ‐Terpinene	1,056	0.65
Terpinolene	1,086	0.66
Linalool	1,088	7.13
trans‐Pinocarvone	1,135	0.27
Terpinen‐4‐ol	1,173	0.32
α‐Terpineol	1,188	4.74
Methyl chavicol	1,194	0.4
Nerol	1,223	0.14
Linalyl acetate	1,252	3.19
Terpinyl acetate	1,343	1.44
Neryl acetate	1,359	0.19
Geranyl acetate	1,379	0.46
trans‐Caryophyllene	1,412	0.16
α‐Humulene	1,446	0.12
Caryophyllene oxide	1,571	0.14
Total		**96.98**

RI, retention indices determined on HP‐5MS capillary column; %, Calculated from TIC data.

**Table 3:** Composition of essential oil of *Thymus Carmanicus*

Compound	RI	Content (rel. %)
α‐Thujene	929.454	0.51
α‐Pinene	936.192	1.31
Camphene	950.74	0.11
Sabinene	978.816	0.32
α‐Terpinene	1,017.22	0.31
*p*‐Cymene	1,024.15	0.61
Limonene	1,028.24	0.5
*cis*‐ Linalool oxide	1,071.61	2.49
*trans*‐Linalool oxide	1,078.62	2.65
Linalool	1,100.16	54.02
α‐Terpineol	1,186.41	1.69
Carvacrol methyl ether	1,250.83	2.01
Thymol	1,285.63	2.67
Carvacrol	1,295.07	19.93
β‐Caryophyllene	1,411.94	1.81
Caryophyllene oxide	1,574.25	0.21
Total		**91.15**

RI, Retention indices determined on HP‐5MS capillary column; %, Calculated from TIC data.

### 3.2 The effect of the antioxidant activity of *M. communis* and *T. carmanicus* essential oils on the physicochemical properties of potato chips

#### 3.2.1 Rancimat test

Foodstuffs that contained oil like potato chips turned rancid over time. This was caused by chemical changes in the fat; in particular, its oxidation was noteworthy. Because of this, determining the oxidation stability of fats and oils in foods has proven to be a useful tool in the quality control of foodstuffs. Antioxidant activity was evaluated by measuring the length of the induction time, as determined by Rancimat test. Rancimat is a rapid automated method that provides reliable information about the oxidation stability of fats and oils (de la Presa‐Owens, Lopez‐Sabater, & Rivero‐Urgell, 1995). The calculated induction period (hr) is shown in Table 4.

**Table 4:** Assessment antioxidant activities about each of the *Thymus carmanicus* and *Myrtus communis* essential oils, as well as BHA and TBHQ synthetic antioxidants by rancimat method

Treatment	Storage time (hr)
Control	11.91^a^ *
*Thymus carmanicus*	12.72^c^
*Myrtus communis*	12.12^ab^
BHA	13.06^b^
TBHQ	38.42^d^

*Different superscripted letters (a‐d) indicate significant differences (*p* < .05).

#### 3.2.2 Peroxide value

Lipid oxidation involves the continuous formation of primary oxidation products; hydroperoxides that are unstable may be decomposed, yielding secondary products (Dobarganes & Velasco, 2002). PV, as an indicator of the initial stages of oxidative change, is one of the most common quality indicators of fats and oils during production and storage (Antolovich et al., 2008). Iodometric titration assay is one of the most frequently used methods for the determination of PV. In this method, a saturated solution of potassium iodide is added to oil samples to react with hydroperoxides. The liberated iodine (I_2_) is then titrated with a standardized solution of sodium thiosulfate and starch as an endpoint indicator (Antolovich et al., 2008).

**Figure 1:**
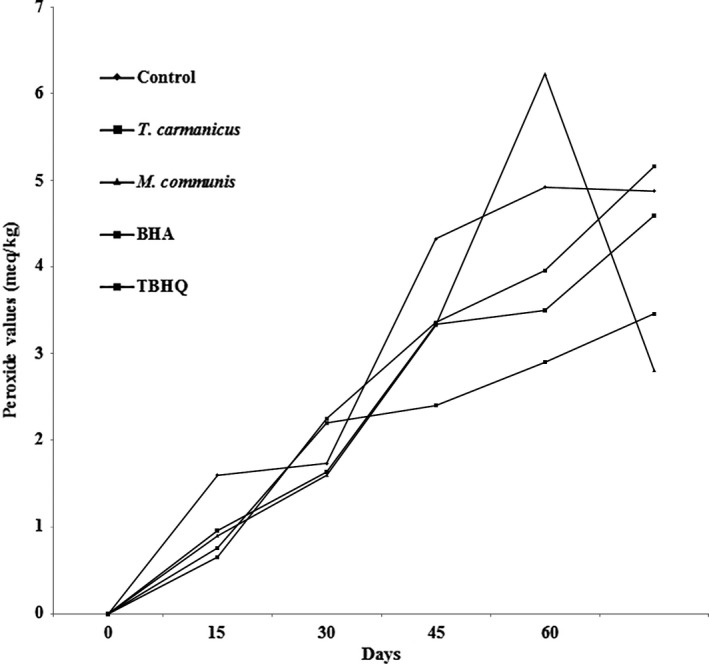
Peroxide values of potato chips containing BHA, TBHQ, *Thymus carmanicus*, and *Myrtus communis* essential oils

Results of PV were analyzed on the first day in different treatments; then, the changes were measured during 60 days of storage. As shown in Figure 1, samples containing *M. communis*,* T. carmanicus*, BHA, or TBHQ showed a significant decrease in PV, as compared to the control sample (0.079).

#### 3.2.3 Thiobarbituric acid (TBA)

**Figure 2:**
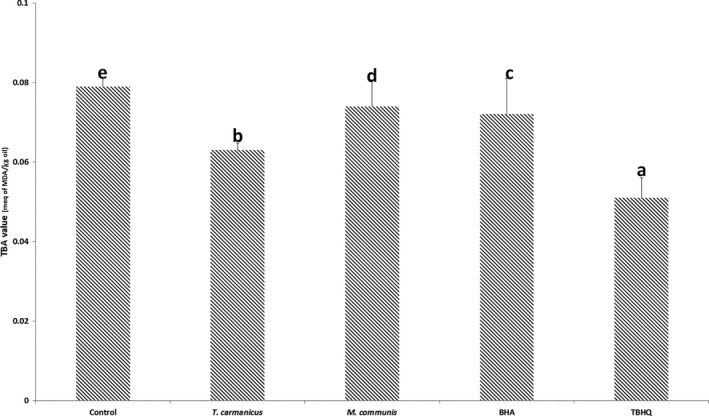
Thiobarbituric acid (TBA) values of potato chips containing BHA, TBHQ, *Thymus carmanicus*, and *Myrtus communis* essential oils

Figure 2 indicates that the addition of the essential oils of *M. communis*,* T. carmanicus*, BHA, or TBHQ had a significant effect on the TBA of the extracted oil from potato chips. *T. carmanicus* was more effective than BHA in preventing the TBA increasing value; it was followed by *M. communis*.

#### 3.2.4 Acid value (AV)

**Figure 3:**
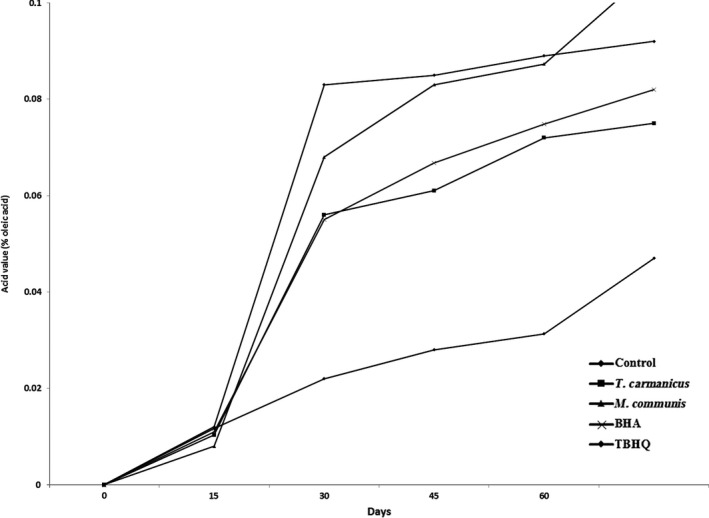
Acid values (% oleic acid) of potato chips containing BHA, TBHQ, *Thymus carmanicus*, and *Myrtus communis* essential oils during 60 days of the storage period

In Figure 3, the curves of AV obtained for fried potato chips during shelf‐life are presented. AV from the extracted oil of samples indicated that the addition of *M. communis*,* T. carmanicus* essential oils, and BHA or TBHQ synthetic antioxidants significantly reduced AV in potato chips. Sunflower oil without any additive (control) produced severe acidity during potato frying; however, the addition of antioxidants, either synthetic (TBHQ and BHA) or natural (*M. communis* and *T. carmanicus*), improved its acid value.

#### 3.2.5 Effects on texture

**Figure 4:**
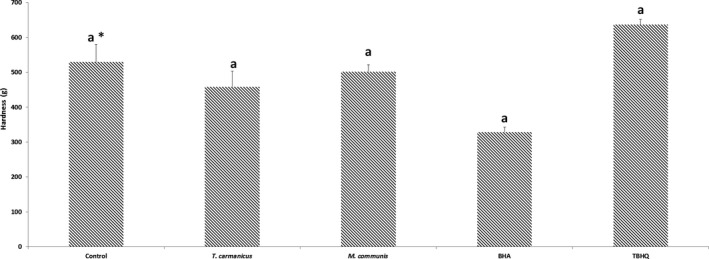
The effects of BHA, TBHQ, *Thymus carmanicus*, and *Myrtus communis* essential oils on the hardness of potato chips

Figure 4 shows hardness results for potato chips with or without treatments. In comparison with the control group, more hardness and less crispness were exhibited by potato chips with TBHQ. The rigidity was decreased significantly with *M. communis*,* T. carmanicus*, or BHA; however, it had no significant effect on the breaking force of potato chips (*p* > .05), as compared with the control sample.

#### 3.2.6 Effects on color

Color of potato chips is a particularly important characteristic for the potato processing industry, and it is stringently related to consumer acceptance (Scanlon, Roller, Mazza, & Pritchard, 1994). Color of fried potatoes has been usually measured in units L*a*b*. L* is the luminance or lightness component, which ranges from 0 to 100 (Papadakis, Abdul‐Malek, Kamdem, & Yam, 2002). The comparison of L* values indicated that samples containing BHA or TBHQ had a significantly whiter color (higher L* value), as compared to control (*p* < .05); in the samples containing *M. communis* or *T. carmanicus,* essential oils improved the lightness of potato chips, showing no significant difference with the control (Table 5). Indeed, the lightness value (L*) was 54.65 for the control chips, whereas 57.39 and 55.96 were found for potato chips fried by the enriched sunflower oil in the case of *T. carmanicus* or *M. communis* essential oil, respectively (Table 5). It may be inferred that the volatility of essential oils led to the evaporation of heated oil in the earlier stages of frying. Unlike L*, BI did not follow a defined rule, indicating the reason for the lower dark color in synthetic antioxidants; in addition, the dark color was effective in the BI increase.

**Table 5:** Lightness (L*), color difference (**∆**E), and browning index (BI) of potato chips

Treatment	Browning index (BI)	∆E	L*
Control	85.98^c^	0	54.65^a^*
*Thymus carmanicus*	72.21^ab^	14.84	57.39^a^
*Myrtus communis*	88.84^c^	8.28	55.96^a^
BHA	68.54^a^	99.41	62.41^b^
TBHQ	79.42^bc^	74.95	64.37^b^

Different superscripted letters (a‐d) indicate significant differences (*p* < .05).

## 4 DISCUSSION

Twenty‐six components, accounting for 96.98% of the total composition, were identified for the *M. communis* essential oil (Table 2). The composition of the *M. communis* essential oil predominantly included α‐pinene (41.55%) and 1,8‐cineole (32.24%), linalool (7.13%), and α‐terpineol (4.74%), as identified by the major components. According to the chromatogram, 16 compounds representing 91.15% of *T. carmanicus* essential oil were isolated. According to Table 3, linalool (54.02%) and carvacrol (19.93%) as the main constituents accounted for 73.95% of the total compounds of *T. carmanicus*. Our findings were comparable to the results obtained by Ghasemi et al. (2011); in their study, α‐pinene (31.8%), 1,8 cineole (24.6%), limonene (14.8%), linalool (8.3%), and α‐terpineol (4.8%) were the main components of the *M. communis* essential oil (19). Other studies have determined carvacrol, p‐cymene, γ‐terpinene, and thymol as the major constituents of *T. carmanicus* (Nejad Ebrahimi, Hadian, Mirjalili, Sonboli, & Yousefzadi, 2008; Safaei‐Ghomi et al., 2009). The main cause of these variations in the chemical composition of essential oils could be related to the origin, the environmental conditions, the developmental stage of collected plant materials, and the extraction method.

The results of Rancimat test showed that the control sample without any antioxidants had the lowest induction period (11.91 hr); this was followed by *T. carmanicus* (12.72 hr), *M. communis* (12.12 hr), and BHA (13.06 hr). The greatest shelf‐life was found in the TBHQ group (38.42 hr). As a result, the addition of each of *T. carmanicus* or *M. comminnus* essential oils, as well the synthetic antioxidants (BHA and TBHQ), could increase the shelf‐life of sunflower oil significantly (*p* < .05).

The rancidity development of the control group could be due to lipid oxidation (Hashemipour, Kermanshahi, Golian, & Veldkamp, 2013). The increase in the oxidative stability of the oils extracted from chips fried in oil containing *M. communis* suggested the role of phenolic compounds in protecting against oxidation. In addition, bioactive compounds isolated from Thymus species including thymol and carvacrol could have a considerable antioxidant activity (Giorge et al., 2010). Our findings agreed with the results reported by Rababah, Feng, and Yang (2012), who found that natural antioxidants grape seed extract and green tea extract (either at the 1000 ppm level) were effective in preventing the rancidity development during 60 days of storage. A study showed that a mixture of 400 ppm methanolic extract and mango seed kernel oil increased the oxidative stability of sunflower oil, both at the ambient and frying temperatures, as well as the quality characteristic of potato chips. This effect was due to the phenolic compounds of mango seed kernel oil, which had antioxidant activities on sunflower oil and potato chips (Abdalla, Darwish, Ayad, & El‐Hamahmy, 2007).

According to Figure 1, the samples containing *M. communis* had the highest slope of PV, while TBHQ had the lowest slope. The samples containing natural antioxidants *M. communis* or *T. carmanicus* had a lower slope than BHA. Our findings were in agreement with the results of Rababah et al. (2012), who found the PV values of the potato chips enriched with grape seed extracts or green tea extracts, which contained a large amount of antioxidant compounds, were lower than those of the control during 90 days of storage. A study showed that the addition of *Bunium persicum* essential oil to the crude soybean oil could decrease the PV of oil in the accelerated condition at 60°C by antioxidant activity; consequently, it was able to reduce the oxidation rate (Shahsavari, Barzegar, Sahari, & Naghdibadi, 2008). From these findings, it could be assumed that the improvement of stability against oxidation is due to the presence of some components in the essential oils, such that the antioxidant activity of thymol, carvacrol, α‐pinene, and linalool could be observed.

Designed for the measurement of the secondary products of oxidation, the TBA test is now one of the most commonly used methods to measure the oxidative deterioration of fat‐containing foods (Kishida et al., 1993). The test is based on the color product resulting from the reaction of TBA with malondialdehyde (MDA); it is generated in the oxidized lipids, which is measured spectrophotometrically at its absorption maximum at 530–535 nm (Antolovich et al., 2008). As shown in Figure 2, these results were in agreement with the findings of Allam and El‐Sayed (Allam & El‐Sayed, 2004). In their study, potato chips were prepared using olein oil enriched by TBHQ, vitamin A (retinyl palmitate), vitamin E (tocopherol), or ascorbyl palmitate (vitamin C); the results showed that TBA values of potato chips fried using vitamin C or A were lower than those of control (Allam & El‐Sayed, 2004). In thyme‐treaded nuggets, the antioxidant effect of essential oil resulted in the improvement of the oxidative stability and the reduction in TBA value within the 6 months of storage (Ganjali Dashti, Mirlohi, Ganjali Dashti, Jafari, & Bahreini Esfahani, 2015).

As illustrated in Figure 3, samples containing *M. communis* represented a decrease in AV by a significant difference, as compared to the control; however, samples containing *T. carmanicus*, BHA, or TBHQ showed a decrease in AV by no significant difference. As shown in Figure 3, the samples containing *M. communis* and control samples had the highest increasing slope of acidity and TBHQ had the lowest slope. The results obtained by Allam and El‐Sayed (2004) showed that fried potato chips in the oil containing vitamins A and C had a lower AV value during the frying period, as compared to the control.

The texture is one of the most important attributes for evaluating the quality of fried potato chips, and consumers' satisfaction strongly depends on the chips' texture. A recommended assay to evaluate the texture of fried potato chips is the puncture test, which measures the maximum breaking force as an indication of crispness (Pedreschi, Segnini, & Dejmek, 2004).

As can be seen in Table 5, based on the values of browning index (BI), the samples containing *T. carmanicus* essential oil and BHA had a significantly lower BI index, which was 72.21 and 68.54, respectively, as compared to the control group (85.98); but the samples containing *M. communis* essential oil had a high BI (88.84) by no significant difference, as compared to the control one. In the TBHQ samples, a lower BI (79.42), as compared to the control, was seen, with no significant difference to that one (*p* > .05). Minimum and maximum BI values were obtained for the samples containing BHA and *M. communis, which were* 68.54 and 88.84, respectively (Table 5). In a study conducted by Che Man and Tan, the effects of oleoresin rosemary or sage extract, by antioxidant activity, on palm olein during deep‐fat frying of potato chips were evaluated. The sensory evaluation of fried potato chips showed that there was no significant difference in terms of flavor, odor, and texture (Che Man & Tan, 1999).

## 5 CONCLUSION

The main aim of this study was to enrich the frying oil with *M. communis* leaf and *T. carmanicus* aerial parts essential oils to produce a harmless potato chips product. Findings of the present investigation demonstrated that potatoes fried in the oil without any additives (control) had the lowest quality and storage stability; it was increased by the addition of antioxidants to BHA, TBHQ, *M. communis,* or *T. carmanicus* essential oils. As described, essential oils could prevent oxidation and undesirable changes, with no effect on the sensory properties.

## CONFLICT OF INTEREST

The authors declare no conflict of interest.
